# Glutamine synthetase sustains cortical circuit development via mTOR-mediated astrocyte maturation

**DOI:** 10.1093/procel/pwaf112

**Published:** 2026-01-02

**Authors:** Pifang Gong, Xiaoli Chen, Wei Cong, Wentong Hong, Yitong Liu, Guibo Qi, Xuan Song, Zhenru Wang, Zhanmeng Leng, Shumin Duan, Jun Gao, Woo-Ping Ge, Song Qin

**Affiliations:** Shanghai Stomatological Hospital and Department of Histoembryology, School of Basic Medical Sciences, State Key Laboratory of Brain Function and Disorders and MOE Frontiers Center for Brain Science, Fudan University, Shanghai 200032, China; Institute of Biophysics, Chinese Academy of Sciences, Beijing 100101, China; Changping Laboratory, Beijing 102206, China; School of Industry-education Integration, University of Chinese Academy of Sciences, Beijing 100190, China; Institute for Translational Brain Research, MOE Frontiers Center for Brain Science, Fudan University, Shanghai 200032, China; School of Basic Medical Sciences, Bengbu Medical University, Bengbu 230061, China; Shanghai Stomatological Hospital and Department of Histoembryology, School of Basic Medical Sciences, State Key Laboratory of Brain Function and Disorders and MOE Frontiers Center for Brain Science, Fudan University, Shanghai 200032, China; Shanghai Stomatological Hospital and Department of Histoembryology, School of Basic Medical Sciences, State Key Laboratory of Brain Function and Disorders and MOE Frontiers Center for Brain Science, Fudan University, Shanghai 200032, China; Shanghai Stomatological Hospital and Department of Histoembryology, School of Basic Medical Sciences, State Key Laboratory of Brain Function and Disorders and MOE Frontiers Center for Brain Science, Fudan University, Shanghai 200032, China; Shanghai Stomatological Hospital and Department of Histoembryology, School of Basic Medical Sciences, State Key Laboratory of Brain Function and Disorders and MOE Frontiers Center for Brain Science, Fudan University, Shanghai 200032, China; Shanghai Stomatological Hospital and Department of Histoembryology, School of Basic Medical Sciences, State Key Laboratory of Brain Function and Disorders and MOE Frontiers Center for Brain Science, Fudan University, Shanghai 200032, China; Department of Neurobiology, Key Laboratory of Medical Neurobiology of Ministry of Health of China, Zhejiang University School of Medicine, Hangzhou 310058, China; Shanghai Stomatological Hospital and Department of Histoembryology, School of Basic Medical Sciences, State Key Laboratory of Brain Function and Disorders and MOE Frontiers Center for Brain Science, Fudan University, Shanghai 200032, China; Changping Laboratory, Beijing 102206, China; Chinese Institute for Brain Research, Beijing 102206, China; Beijing Institute for Brain Research, Chinese Academy of Medical Sciences and Peking Union Medical College, Beijing 102206, China; Shanghai Stomatological Hospital and Department of Histoembryology, School of Basic Medical Sciences, State Key Laboratory of Brain Function and Disorders and MOE Frontiers Center for Brain Science, Fudan University, Shanghai 200032, China

Dear Editor,

The developing cerebral cortex requires precise metabolic regulation to support neurogenesis and circuit formation (Belanger et al., 2011; Namba et al., 2021). Glutamine synthetase (GS), which catalyzes glutamate-to-glutamine conversion, sustains neurotransmitter recycling and nitrogen homeostasis (Tani et al., 2014). Human *GLUL* mutations cause lethal neurodevelopmental disorders (Häberle et al., 2005), and GS deletion in mice leads to postnatal death (He et al., 2010), underscoring its essential role. While adult cortical GS deficiency triggers neurodegeneration (Zhou et al., 2019), its function in early cortical development remains elusive, despite evidence linking astrocytic metabolism to circuit maturation (Chung et al., 2015; Pekny et al., 2016).

Spatiotemporal analysis revealed high GS expression in embryonic neural stem cells (NSCs) within the ventricular/subventricular zone, transitioning to S100β^+^ astrocytes postnatally (Figs. 1A–D and S1A–C). GS enzymatic activity followed a biphasic pattern, peaking during early postnatal stages coincident with astrocyte maturation (Fig. S1D), suggesting stage-specific roles in cortical development.

**Figure 1.**
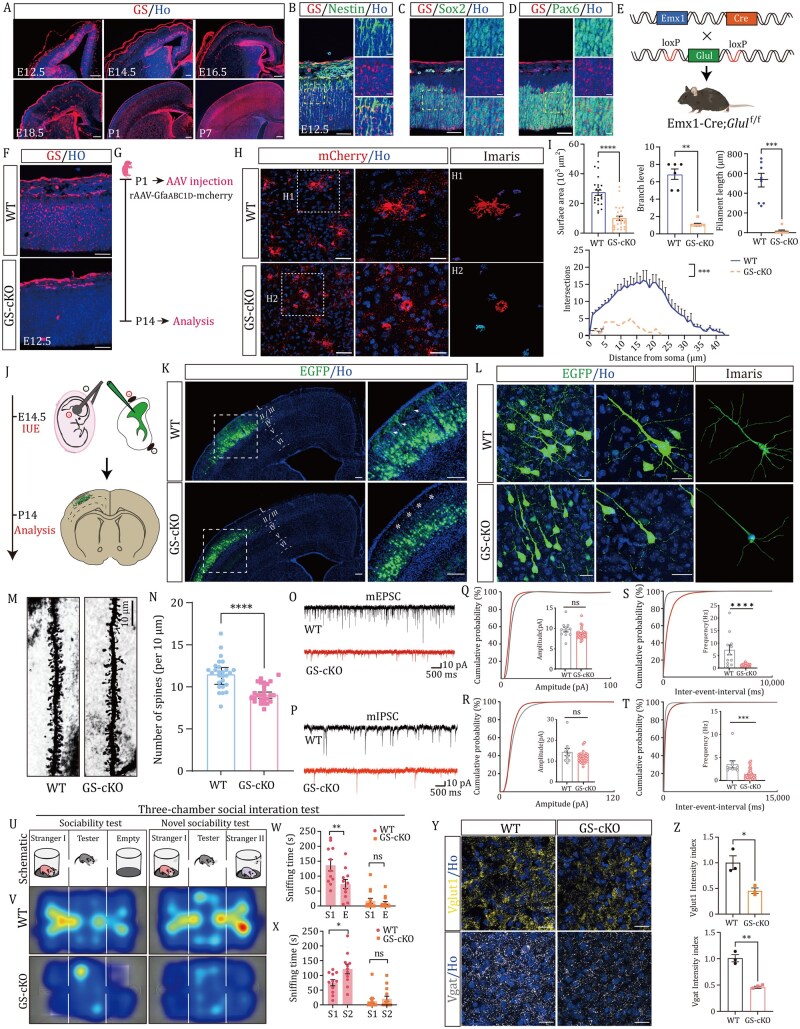
**Glutamine synthetase regulates cortical astrocyte maturation and synaptic development**. (A) Representative images of cortical sections stained for glutamine synthetase (GS) at E12.5, E14.5, E16.5, E18.5, and postnatal days P1 and P7. Scale bars: 200 μm. (B-D) Representative images of GS co-stained with antibodies against Nestin (B), Sox2 (C), or Pax6 (D) in the cortex at E12.5. Yellow dashed outlines indicate regions enlarged on the right. Scale bars: 50 μm (left); 20 μm (right). (E) Breeding strategy for generating GS conditional knockout (cKO) mice. (F) Immunofluorescent staining of GS in wild-type (WT) and GS-cKO mice at E12.5. Scale bars: 50 μm. (G) Schematic and timeline for selective astrocyte labeling in the postnatal cortex. (H) Representative mCherry^+^ astrocytes in P14 WT and GS-cKO cortices. Enlarged insets show reconstructed astrocytes (H1, H2) (Scale bars: 50 μm; 25 μm for H1–H2) (≥20 astrocytes from 3–4 mice per genotype, *n *≥ 3 mice). (I) Quantification of astrocyte morphology, including surface area, branch level, total process length, and Sholl analysis (≥20 astrocytes from 3 to 4 mice per genotype, *n *≥ 3 mice). (J) Schematic of the experimental approach. Embryos were electroporated at E14.5, and brain sections were collected at P14. (K) Representative images of P14 brain sections following in utero electroporation (IUE) with a pCAG-EGFP plasmid at E14.5. White rectangles indicate regions enlarged on the right. Arrowheads highlight complex dendritic branching in the wild-type cortex, while asterisks denote sparse dendritic branching in GS-cKO mice. Scale bars: 200 μm. (L) Representative images of EGFP^+^ pyramidal neurons reconstructed using Imaris software. (M) Representative images of dendritic spines in WT and GS-cKO mice at P28. Scale bars: 10 μm. (N) Quantification of spine density per 10 μm on secondary dendrites (10 neurons per animal, 3 animals per genotype). (O and P) Representative traces of whole-cell mEPSC (O) and mIPSC (P) recordings from layer II/III pyramidal neurons in acute M1 slices of P28 WT and GS-cKO mice. (Q and R) Cumulative distributions of the amplitude of mEPSC (Q) and mIPSC (R) in P28 WT and GS-cKO pyramidal neurons. (S and T) Cumulative distributions of the inter-event interval of mEPSC (S) and mIPSC(T) in P28 WT and GS-cKO pyramidal neurons. (U) Schematic of the three-chamber sociability test apparatus. (V) Representative heatmaps of movement traces from WT and GS-cKO mice during the three-chamber sociability and novel sociability tests. (W and X) Quantification of sniffing time during the sociability test (W) and novel sociability test (X) (WT: *n *= 18 mice; GS-cKO: *n *= 15 mice). (Y) Immunofluorescent images of excitatory (VGLUT1) and inhibitory (VGAT) synapses in layer II/III neurons of P28 WT and GS-cKO mice. Scale bars: 25 μm. (Z) Quantification of VGLUT1 and VGAT intensity indices in P28 WT and GS-cKO mice (*n *= 3 mice). Statistical significance was determined using an unpaired two-tailed Student’s *t*-test (N, P, Q, S, T, W, X, Z) or an unpaired two-tailed Mann–Whitney test (I). *P *< 0.05, *P *< 0.01, *P < 0.001, **P < 0.0001; ns, not significant. Nuclei were counterstained with Hoechst 33342 (Ho; blue).

To dissect the *in vivo* function of GS, we generated cortex-specific GS knockout (GS-cKO) mice by crossing ­*Glul^flox^*^/^^*flox*^ mice with *Emx1-Cre* mice (Fig. 1E). This strategy induces gene ablation in radial glial progenitors from embryonic day 10.5 onward while preserving postnatal viability, thereby enabling examination of GS function across developmental stages (Gorski et al., 2002). Efficient GS deletion was confirmed in both embryonic and postnatal forebrains (Figs. 1F and S1E–G). By postnatal day 14 (P14), GS-cKO mice exhibited reduced brain size and ­cortical thickness (Fig. S1H–K), accompanied by notable astrocytic morphological abnormalities, as revealed by AAV2/9-GFAP-mCherry labeling (Fig. 1G–I). In contrast, embryonic neurogenesis and neuronal migration remained largely intact (Figs. 1J, 1K, and S2A–I). Although early GS loss mainly impaired astrocyte maturation, by P28, the cortex of GS-cKO mice displayed pronounced astrocyte activation, characterized by robust GFAP upregulation, cellular hypertrophy, and thickened astrocytic processes, indicative of reactive gliosis (Fig. S3A–C). Notably, dietary glutamine supplementation partially restored astrocyte maturation, as evidenced by improved morphological complexity and enhanced process elaboration (Fig. S4A–C), highlighting the essential role of GS-derived glutamine in supporting astrocytic structural development.

GS deficiency severely impaired dendritic growth, spine density, and synaptogenesis, as shown by EGFP electroporation, Golgi staining, and synaptic marker analysis (Figs. 1M, 1N, 1Y, 1Z and S4D). Electrophysiological recordings confirmed reduced miniature excitatory and inhibitory synaptic activity (Fig. 1O–T). Remarkably, glutamine ­supplementation restored synaptic density, indicating metabolite dependence of GS-mediated circuit maturation (Fig. S4E–H). Consistently, GS-cKO mice displayed behavioral abnormalities in motor coordination (Figs. S5A–E, S5G, and S5H) and social interaction (Figs. 1U–X and S5F), paralleling behavioral deficits in autism and epilepsy models (Varghese et al., 2017; Sharma et al., 2018).

Astrocyte development was prominently disrupted in GS-cKO mice. GS deficiency reduced S100β^+^, Aldh1L1^+^, Sox2^+^, and Sox9^+^ cell numbers (Fig. 2A–H) without increasing apoptosis (Fig. S6A and S6B). Single-nucleus RNA sequencing (snRNA-seq) of P7 cortex identified major cell types but showed no change in excitatory neuron or astrocyte proportions between genotypes (Fig. 2I–K). However, analysis of astrocyte-specific gene expression revealed distinct alterations, with a downregulation of Blbp (a key regulator of astrocyte maturation) and an upregulation of ApoE (a lipid transporter implicated in neuroinjury response), respectively; in contrast, the glutamate transporter Slc1a3 (GLAST) remained unchanged in GS-cKO astrocytes (Figs. 2L, S6H, S6J, and S6M). These changes emerged by P1 (Fig. S6C–F) and were confirmed by reduced Blbp protein and increased *ApoE* mRNA via immunostaining and *in situ* hybridization (Fig. S6H–O).

**Figure 2.**
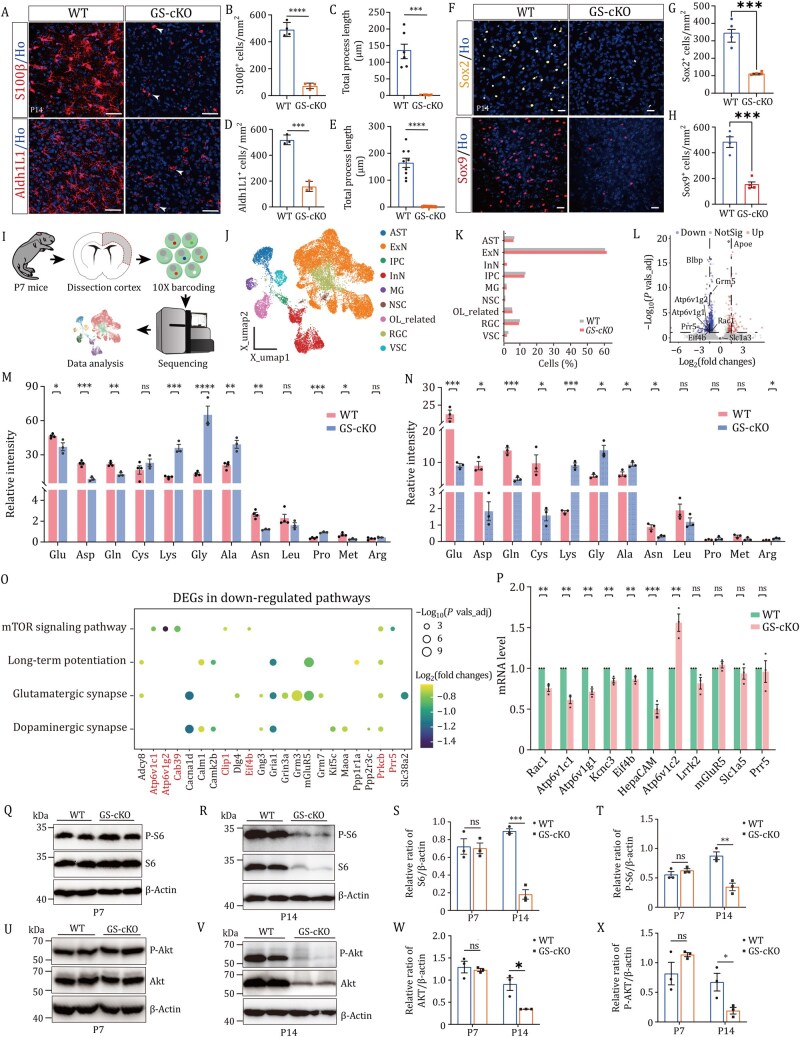
**GS deficiency suppresses mTOR signaling and disrupts amino acid homeostasis in cortical astrocytes**. (A) Immunofluorescent staining of S100β and Aldh1L1 in P14 WT and GS-cKO mice. Arrowheads indicate unbranched astrocytes in the GS-cKO cortex (Scale bars: 50 μm). (B–E) Quantification of S100β^+^ or Aldh1L1^+^ astrocytes (≥20 cells per mouse) and astrocyte complexity (based on total processes) from 3 to 4 mice per genotype (*n *≥ 3 mice). (F) Representative images of P14 WT and GS-cKO brain sections stained for Sox2 (orange) or Sox9 (red) (Scale bars: 25 μm). (G and H) Quantification of Sox2^+^ or Sox9^+^ cells in the cortex at P14 (*n *≥ 3 mice). (I) Schematic of snRNA-seq using cortex samples from WT and GS-cKO mice at P7. (J) t-SNE plot showing broad cell-type clustering based on transcriptomic profiles. (K) Histogram of the percentage of different cell types. AST, astrocytes; ExN, excitatory neurons; IPC, intermediate precursor cells; InN, inhibitory neurons; MG, microglia; NSC, neural stem cells; OL_related, oligodendrocytes and OPCs; RGC, radial glial cells; VAC, vascular endothelial cells. (L) Differentially expressed genes in the astrocyte cluster, highlighting astrocyte markers and mTOR pathway-related genes. (M and N) Relative amino acid levels in cortical tissues from WT and GS-cKO mice at P14 (M) or P28 (N) (*n *≥ 3 mice). (O) Dot plot of differentially expressed genes in astrocytes from GS-cKO mice compared to WT, highlighting KEGG pathway enrichment. (P) qRT-PCR analysis of candidate genes based on RNA-seq data from WT and GS-cKO mice at P14 (*n *= 3 mice). (Q and R) Western blot analysis of S6 and phosphorylated S6 (p-S6) in the cortex of WT and GS-cKO mice at P7 (Q) and P14 (R) (*n *= 3 mice). (S and T) Quantification of S6 (S) and p-S6 (T) protein levels normalized to β-actin (*n *= 3 mice). (U and V) Western blot analysis of AKT and phosphorylated AKT (p-AKT) in the cortex of WT and GS-cKO mice at P7 (U) and P14 (V) (*n *= 3 mice). (W and X) Quantification of AKT (W) and p-AKT (X) protein levels normalized to β-actin (*n *= 3 mice). Statistical significance was determined using an unpaired two-tailed Student’s *t*-test. *P < 0.05, **P < 0.01, ***P < 0.001, ****P < 0.0001; ns, not significant.

Metabolically, GS loss reduced glutamine, glutamate, aspartate, and asparagine, while elevating glycine and alanine (Fig. 2M and 2N). snRNA-seq revealed selective suppression of the mTOR pathway in astrocytes (Fig. 2O and 2P). Western blotting confirmed decreased phosphorylation of S6 (mTORC1) and AKT (mTORC2), with key mTOR pathway genes (*Atp6v1c1*, *Atp6v1g2*, *Eif4b*) downregulated (Fig. 2P–X). Thus, GS deficiency disrupts cortical amino acid homeostasis and suppresses astrocytic mTOR signaling, likely underlying the observed astrocyte dysfunction and neuronal deficits.

Our findings establish GS as a key regulator of cortical circuit maturation through astrocyte metabolic programming. GS expression shifts from NSCs to astrocytes during cortical expansion, indicating stage-specific roles. Neurogenesis remains unaffected, likely due to maternal or placental glutamine supply (Wu et al., 2015), but postnatal GS deficiency profoundly impairs astrocyte maturation and suppresses mTOR signaling, suggesting that mTOR inactivation is central to the observed developmental deficits.

As a metabolic sensor, mTOR critically depends on intracellular amino acid availability. Glutamine and its metabolites (e.g., α-ketoglutarate and arginine) not only directly activate mTORC1 but also fuel mTOR-dependent anabolic processes (Jewell et al., 2015). Thus, GS loss removes both signaling input and metabolic support for mTOR, leading to selective pathway impairment. In contrast, the Hippo/YAP pathway, although sensitive to energy stress (Sheng et al., 2024), lacks a glutamine-sensing mechanism and is therefore less affected. This specificity explains why mTOR signaling is preferentially disrupted in GS-deficient astrocytes.

mTOR suppression likely compromises astrocytic metabolic support, reducing protein synthesis and precursor provision for nucleotide and neurotransmitter production. Such deficits during critical developmental windows disrupt synaptogenesis and circuit refinement, offering a mechanistic link between GS–mTOR dysregulation and neurodevelopmental disorders, such as specific autism spectrum disorder subtypes. Meanwhile, upregulation of ApoE may initially serve as a compensatory response to metabolic stress but, when prolonged, can lead to lipid dysregulation, toxic protein accumulation, and increased susceptibility to neurodegenerative conditions, consistent with APOE ε4-associated Alzheimer’s disease (Zheng and Wang, 2025).

Our observations align with the previously reported *Glul* conditional knockout model (Zhou et al., 2019), which also exhibited seizures, motor deficits, and reactive astrogliosis. Although our study documents cortical thinning earlier and more quantitatively, careful review of their data ­suggests similar trends. Differences in phenotypic presentation likely reflect variations in timing and targeting strategies; nevertheless, both models converge on severe astrogliosis by the fourth postnatal week, highlighting GS loss as a potent driver of neuroinflammatory pathology.

In conclusion, this study uncovers an essential role for GS in postnatal brain development and reveals how a metabolic enzyme can regulate cell-specific gene expression, protein translation, and morphology. These insights offer new therapeutic perspectives for treating neurological disorders involving astrocytic dysfunction and impaired cortical connectivity, such as epilepsy and autism.

## Footnotes

We thank the members of the Qin laboratory for their valuable suggestions and comments. This work was supported by grants from the National Natural Science Foundation of China (Nos. 31871477, 32170971), the Natural Science Foundation of Shanghai (No. 18ZR1403800) awarded to S.Q.; as well as grants from the CAMS Innovation Fund for Medical Sciences (CIFMS, No. 2024-I2M-ZD-012), the STI2030-Major Projects (No. 2022ZD0204700), and the National Natural Science Foundation of China (No. 32170964) awarded to W.-P.G.

The authors declare that they have no competing interests, financial or non-financial, that could be perceived as influencing the work reported in this manuscript.

All animal procedures were approved by the Fudan University Institutional Animal Care and Use Committee and performed in accordance with institutional and national guidelines.

All authors have provided informed consent for participation and confirmed their agreement to publish this work.

The datasets used and/or analyzed during the current study are available from the corresponding author upon reasonable request.

S.Q., W.P., J.G., and S.D. conceived the project. S.Q., W.P., and P.G. designed experiments. P.G. performed most of the experiments. X.C. performed single-cell analysis. W.C. ­performed electrophysiological experiments. W.H., Y.L., G.Q., X.S., Z.W., and Z.L. assisted with the experiments and helped to analyze the data. P.G. and S.Q. wrote the article.

## Supplementary Material

pwaf112_Supplementary_Data

## References

[pwaf112-B1] Belanger M , Allaman I, Magistretti PJ. Brain energy metabolism: focus on astrocyte-neuron metabolic cooperation. Cell Metab 2011;14:724–738. 22152301 10.1016/j.cmet.2011.08.016

[pwaf112-B2] Chung WS , Allen NJ, Eroglu C. Astrocytes control synapse formation, function, and elimination. Cold Spring Harb Perspect Biol 2015;7:a020370. 25663667 10.1101/cshperspect.a020370 PMC4527946

[pwaf112-B3] Gorski JA , Talley T, Qiu M et al. Cortical excitatory neurons and glia, but not GABAergic neurons, are produced in the Emx1-expressing lineage. J Neurosci 2002;22:6309–6314. 12151506 10.1523/JNEUROSCI.22-15-06309.2002 PMC6758181

[pwaf112-B4] Häberle J , Görg B, Rutsch F et al. Congenital glutamine deficiency with glutamine synthetase mutations. N Engl J Med 2005;353:1926–1933. 16267323 10.1056/NEJMoa050456

[pwaf112-B5] He Y , Hakvoort TB, Vermeulen JL et al. Glutamine synthetase deficiency in murine astrocytes results in neonatal death. Glia 2010;58:741–754. 20140959 10.1002/glia.20960

[pwaf112-B6] Jewell JL , Kim YC, Russell RC et al. Metabolism. Differential regulation of mTORC1 by leucine and glutamine. Science 2015;347:194–198. 25567907 10.1126/science.1259472 PMC4384888

[pwaf112-B7] Namba T , Nardelli J, Gressens P et al. Metabolic regulation of neocortical expansion in development and evolution. Neuron 2021;109:408–419. 33306962 10.1016/j.neuron.2020.11.014

[pwaf112-B8] Pekny M , Pekna M, Messing A et al. Astrocytes: a Central element in neurological diseases. Acta Neuropathol 2016;131:323–345. 26671410 10.1007/s00401-015-1513-1

[pwaf112-B9] Sharma SR , Gonda X, Tarazi FI. Autism spectrum disorder: classification, diagnosis and therapy. Pharmacol Ther 2018;190:91–104. 29763648 10.1016/j.pharmthera.2018.05.007

[pwaf112-B10] Sheng X , Xia Z, Yang H et al. The ubiquitin codes in cellular stress responses. Protein Cell 2024;15:157–190. 37470788 10.1093/procel/pwad045 PMC10903993

[pwaf112-B11] Tani H , Dulla CG, Farzampour Z et al. A local glutamate-glutamine cycle sustains synaptic excitatory transmitter release. Neuron 2014;81:888–900. 24559677 10.1016/j.neuron.2013.12.026 PMC4001919

[pwaf112-B12] Varghese M , Keshav N, Jacot-Descombes S et al. Autism spectrum disorder: neuropathology and animal models. Acta Neuropathol 2017;134:537–566. 28584888 10.1007/s00401-017-1736-4 PMC5693718

[pwaf112-B13] Wu X , Xie C, Zhang Y et al. Glutamate-glutamine cycle and exchange in the placenta-fetus unit during late pregnancy. Amino Acids 2015;47:45–53. 25399054 10.1007/s00726-014-1861-5

[pwaf112-B14] Zheng Q , Wang X. Alzheimer’s disease: insights into pathology, molecular mechanisms, and therapy. Protein Cell 2025;16:83–120. 38733347 10.1093/procel/pwae026 PMC11786724

[pwaf112-B15] Zhou Y , Dhaher R, Parent M et al. Selective deletion of glutamine synthetase in the mouse cerebral cortex induces glial dysfunction and vascular impairment that precede epilepsy and neurodegeneration. Neurochem Int 2019;123:22–33. 30053506 10.1016/j.neuint.2018.07.009 PMC8261904

