# Supplementary material for: Glutamine synthetase sustains cortical circuit development via mTOR-mediated astrocyte maturation
Source: Protein Cell. 2026 Jan 2;17(6):573–7. doi: 10.1093/procel/pwaf112 (PMC13225726; doi:10.1093/procel/pwaf112)
Supplement: pwaf112_Supplementary_Data [file pwaf112_supplementary_data.zip › pwaf112_Supplementary_Data/all_supplemetary_file.pdf]

Materials and methods

Animals

Glul-floxed (Stock No. 029827, Jackson Laboratory), *Emx1-Cre* (Stock No. 005628, Jackson Laboratory) mice were housed under a 12-h light/dark cycle at 23 ± 2°C with ad libitum access to food/water. Embryonic day 0.5 (E0.5) was defined as midday on the day of vaginal plug detection. Littermates without Cre alleles served as controls. All procedures followed protocols approved by the Animal Care Committee of Shanghai Medical College, Fudan University.

Tissue preparation

Mice were anesthetized with sodium pentobarbital (50 mg/kg) and transcardially perfused with ice-cold PBS followed by 4% paraformaldehyde (PFA). Brains were post-fixed overnight in 4% PFA at 4°C, cryoprotected in 30% sucrose for 48 h, embedded in OCT compound, and coronally sectioned (14 or 40 μm) using a Leica cryostat.

Immunocytochemistry

Cryosections were incubated in antigen retrieval buffer at 80°C for 20 min, then washed three times with PBS. Next, brain sections were permeabilized with 0.3% Triton X-100 for 30 min and blocked with 5% bovine serum albumin for 1–2 h at room temperature. Primary antibodies were applied overnight at 4°C. The following day, sections were washed with PBS containing 0.1% Triton X-100 and incubated with species-specific secondary antibodies for 1 h at room temperature. Finally, sections were stained with Hoechst 33342 (1 μg/mL) in PBS for 15 minutes and mounted using VECTASHIELD antifade mounting medium. The primary and secondary antibodies used are listed in Supplementary Table 1.

Nissl staining

Coronal brain sections were stained with Cresyl Violet using a Nissl Staining Kit (Solarbio) per manufacturer guidelines. Sections were incubated in Cresyl Violet solution at 56°C for 60 min, rinsed in deionized water, and differentiated in Nissl Differentiation Solution for 1 min. Tissues were dehydrated through an ethanol series (70%, 90%, 100%; 5 min each), cleared in xylene (15 min), and mounted with Neutral Balsam. Images of cortical layers were acquired using a bright-field microscope (EVOS™ M7000) at 20× magnification.

Golgi staining

Mice were anesthetized and transcardially perfused with ice-cold PBS followed by 4% paraformaldehyde. Brains were processed using the FD Rapid GolgiStain™ Kit (FD Neuro Technologies): tissues were immersed in staining solution for 14 days, protected

from light, and sectioned coronally at 100 μm using a cryostat (Leica). Sections were mounted on gelatin-coated slides, air-dried, and imaged at 20× magnification. Dendritic spine density was quantified by counting spines per 10 μm segment across secondary/tertiary dendrites (100× magnification; ImageJ).

In Utero Electroporation (IUE)

In utero electroporation was performed as previously described (Hong et al., 2024). Briefly, pregnant mice at E14.5 were anesthetized with sodium pentobarbital (0.7%, 10 μg/g body weight). After sterilizing the abdominal area with 70% ethanol, the uterine horns were exposed. A 1 μL solution of plasmid DNA (2–3 μg/μL) mixed with Fast Green (10 mg/mL) was microinjected into the lateral ventricles of embryos using polished glass micropipettes. Electrical pulses were then applied by gently clamping the embryos' heads with 5-mm electrode paddles connected to an ECM-830 square-pulse generator (BTX). Five 50-ms pulses at approximately 34 V, with 950-ms intervals, were delivered. Throughout the procedure, the uterus was kept in warm 0.9% saline. After electroporation, the uterus was repositioned in the abdominal cavity, and the incision was sutured. The animal was placed in a temperature-controlled environment until fully recovered and resumed normal activity.

AAV virus injection

Virus injection in newborn pups was performed as previously described (Kim et al., 2014). Briefly, neonatal mice were collected from the cage and anesthetized by cryoanesthesia. A total of 40 nL of AAV solution containing 0.05% Fast Green was injected into the target site using polished glass micropipettes connected to a microinjector pump. The injection site was targeted to the cortex (2.0 mm anterior, 1.8 mm lateral, and 1.2 mm ventral relative to lambda). After the injection, the needle was left in place for 5 min to allow virus diffusion. Once both injections were completed, the pups were placed on a warming pad until they fully recovered and resumed movement.

EdU labeling

EdU (T5648; Sigma-Aldrich) was dissolved in saline at a concentration of 10 mg/mL. P4 pups received an intraperitoneal injection of EdU (5 μg/g body weight) and were collected at P7. Brain tissue was processed as described above. Frozen brain sections were dried at room temperature and washed three times in PBS. Sections were then incubated in a 2 mg/mL glycine solution for 10 minutes, followed by permeabilization with 0.3% Triton X-100 in PBS for 30 minutes. The EdU staining solution was prepared according to the kit instructions and incubated in the dark at 37°C for 1 hour. After staining, immunofluorescence with the desired markers was performed.

Tamoxifen administration

Tamoxifen (T5648; Sigma-Aldrich) was dissolved in a mixture of ethanol and sunflower oil (1:9, v/v) at a concentration of 20 mg/mL. The stock solution was stored at -20°C and diluted to 10 mg/mL before use. P1 mice received an intraperitoneal injection of 50 μg tamoxifen for three consecutive days.

Assay for GS activity

Mouse brain cortex tissue was isolated and homogenized in lysis buffer, followed by centrifugation at 12,000 rpm for 10 min at 4°C. The enzymatic activity of GS was determined using a γ-glutamyltransferase assay. The GS biosynthetic reaction buffer was prepared according to the kit instructions and incubated at 37°C for 30 min. The reaction mixture was then measured using a microplate reader at an absorbance of 540 nm. One unit of enzyme activity was defined as a change of 0.005 in A540 absorbance per gram of tissue per minute.

Western blotting

For Western blot analysis, mouse brain cortex tissue was lysed in RIPA buffer supplemented with protease and phosphatase inhibitors. Protein concentration was measured using the BCA assay (Takara). Equal amounts of protein extract were resolved on a 10%-15% SDS-PAGE gel and transferred to PVDF membranes (Merck-Millipore). Membranes were blocked with 5% milk in TBS containing 0.1% Tween-20 (TBST) for 1 h and subsequently incubated with primary antibodies overnight at 4°C. The primary antibodies used are listed in Supplementary Table 1. The following day, the membranes were incubated with horseradish peroxidase (HRP)-conjugated secondary antibodies for 1 h at room temperature. Anti-mouse or anti-rabbit IgG secondary antibodies were used. Peroxidase activity was detected using the ECL-Plus system. Densitometric analysis of Western blot images was performed using ImageJ software.

TUNEL staining

TUNEL staining was performed using the TUNEL FITC Apoptosis Detection Kit (Vazyme). Briefly, brain sections (14 μm) were fixed in 4% PFA for 30 min, washed three times with PBS, permeabilized with 0.2% Triton X-100 for 20 min, and incubated in 1× equilibration buffer for 20 min at room temperature. After removing excess liquid, TdT enzyme was added and incubated at 37°C for 1 h. Sections were counterstained with 1 μg/mL DAPI, mounted using Fluoromount-G™, and examined under a fluorescence microscope.

Electrophysiology

P26–30 mice were deeply anesthetized with sodium pentobarbital, decapitated, and brains were quickly removed and submerged in ice-cold slicing solution (213 mM Sucrose, 26 mM $NaHCO_3$, 1.25 mM NaH_2PO_4, 2.5 mM KCl, 2 mM $CaCl_2$, 2 mM MgSO4, 10 mM D(+)-glucose, pH 7.4, bubbled with 95% O_2/5% CO_2). Coronal slices (300 μm) were prepared using a Leica vibratome(VT 1200S) and transferred to extracellular ACSF solution (126 mM NaCl, 26 mM $NaHCO_3$, 1.25 mM NaH_2PO_4, 2.5 mM KCl, 2 mM $CaCl_2$, 2 mM $MgSO_4$ and 25mM D(+)-glucose, pH 7.4). bubbled with 95% oxygen, 5% carbon dioxide at 34℃ for 30 min then cooling to room temperature. Slices were transferred to a recording chamber perfused with ACSF (1.2 ml/min) at 34-35℃. L2/3 pyramidal neurons were visualized using an upright Olympus microscope with a ×40 water-immersion objective. Whole-cell recordings were performed using a MultiClamp 700B amplifier and pCLAMP 10 software. Pipettes (5–8 MΩ) were filled with internal solutions: for mEPSCs, 138 mM $CsMeSO_4$, 3 mM CsCl, 10 mM HEPES, 0.2 mM EGTA, 2 mM $MgCl_2$, 2 mM Na_2-GTP, 2 mM QX-314, 0.2% biocytin (pH 7.2), and recording in the presence of 1uM TTX and 50 μM picrotoxin; for mIPSCs, 130 mM CsCl, 10 mM NaCl, 0.8 mM $CaCl_2$,10 mM HEPES, 0.2 mM EGTA, 2 mM $MgCl_2$, 2 mM Na_2ATP, 0.5 mM Na_2GTP, 2mM TEA, 0.2% biocytin (pH 7.2), and recording in the presence of 1uM TTX ,50 μM AP5 and 10 μM CNQX. Both recordings were performed at −70 mV. Signals were digitized using a Digidata 1550B, filtered at 3 kHz, and analyzed with ClampFit (v.10.7).

Behavioral test

Open-field test

Mice were placed in the center of a white acrylic open-field arena (40 × 40 × 40 cm) and allowed to explore freely for 15 min. Locomotor activity (total distance traveled, center vs. periphery time) during the final 10 min was analyzed using EthoVision XT 15 (Noldus).

Self-grooming assay

Mice were placed in an empty cage (22 × 12 cm) and video-recorded for 10 min after a 5-min habituation. Total time spent grooming (face wiping, head/ear scratching, body licking) was quantified.

Juvenile play

P24–P28 mice were habituated for 5 min in a clean cage before introducing an age-/sex-matched unfamiliar juvenile. Social interactions (nose-to-nose/nose-to-anus sniffing, following) were manually scored over 10 min.

Three-chamber social interaction test

The three-chamber social interaction test was conducted in an arena divided into three chambers: left, middle, and right. Two containers were positioned in the corners of the side chambers. The test consisted of three phases. In the first phase, the subject mouse was placed in the center chamber and allowed to freely explore all three chambers, with both containers remaining empty. In the second phase, an unfamiliar mouse (Stranger 1, S1) was placed in one of the side chambers, while the other chamber remained empty. The subject mouse was then allowed to explore both chambers, choosing between interacting with S1 or the empty container. The location of S1 was randomized between the left and right chambers. In the final phase, a second unfamiliar mouse (Stranger 2, S2) was placed in the previously empty chamber, and the subject mouse was allowed to explore both S1 and S2. The time spent sniffing the stranger mice or empty containers was recorded using EthoVision XT13 (Noldus).

Rotarod test

Mice were trained for 4 days (3 trials/day) on an accelerating rotarod (4–40 rpm over 300 sec). Latency to fall and maximum speed achieved were recorded. The apparatus was cleaned with 70% ethanol between trials.

In situ hybridization

In situ hybridization was performed as previously described (Qin et al., 2011) using digoxigenin-labeled riboprobes. Briefly, an 819-bp fragment of mouse *Glul* cDNA was amplified by PCR with primers GS-F (5′-ACCTCCATCCTGTTGCCA-3′) and GS-R (5′-ACCGCATAGGGGTCACAA-3′) and cloned into the *pGEM-T Easy Vector* (Promega). Sense and antisense riboprobes were synthesized by *in vitro* transcription using SP6 or T7 RNA polymerase (Roche), respectively. Hybridization was conducted on 16-μm-thick coronal cryosections of mouse brain tissue.

Amino acid determination

Frozen brain tissue (50 mg) was homogenized in 500 μL 0.6 M perchloric acid and centrifuged at 12,000 × g for 20 min at 4°C. The supernatant was analyzed for free amino acids using liquid chromatography-tandem mass spectrometry (LC-MS/MS) in positive ion mode with selected reaction monitoring (SRM). Transitions (m/z) included: 156 → 110 (histidine), 175 → 70 (arginine), 241 → 74 (cystine), 76 → 30 (glycine), 175 → 74 (asparagine), 106 → 60 (serine), 120 → 74 (threonine), 147 → 84 (lysine), 147 → 130 (glutamine), 148 → 84 (glutamic acid), 90 → 44 (alanine/sarcosine), 116 → 70 (proline), 118 → 72 (valine), 150 → 133 (methionine), 132 → 86 (isoleucine/leucine), 182 → 136 (tyrosine), 166 → 120 (phenylalanine), 205 → 188 (tryptophan), and 134 → 74 (aspartic acid). Optimal collision energies were determined using pure amino acid standards. Data were interpreted using TraceFinder (Thermo Fisher).

qRT-PCR

Total RNA was isolated from the cerebral cortex of P14 mice using TRIzol® reagent (Invitrogen). cDNA was synthesized from 1 μg total RNA using random hexamers and the SuperScript™ III First-Strand Synthesis System (Takara Bio) following the manufacturer's protocol. Quantitative PCR (qPCR) was performed in triplicate 20 μL reactions containing TB Green Premix Ex Taq™ (Takara Bio) on a QuantStudio™ 5 Real-Time PCR System (Applied Biosystems). Thermal cycling conditions: 95°C for 10 min (initial denaturation), followed by 40 cycles of 95°C for 5 sec and 60°C for 34 sec. Melt curve analysis (60°C to 95°C, 0.3°C/sec increment) confirmed primer specificity. Relative gene expression was calculated using the ΔΔCt method normalized to *β-actin* (*Actb*) as an endogenous control. Primer sequences are provided in Supplementary Table 2.

L-glutamine supplementation

The experimental group received drinking water supplemented with 0.8% L-glutamine from the last 2–3 days of gestation until P28 of the offspring. To ensure stability, the L-glutamine solution was freshly prepared and replaced daily. At P28, brain tissues were collected, processed for cryosectioning or paraffin embedding, and subjected to immunofluorescence staining and quantitative analysis.

Isolation and culture of cortical astrocytes

Cortices were dissected from P0 mouse brains and enzymatically dissociated using a protease/nuclease mixture in high-glucose DMEM (Gibco) to obtain a single-cell suspension. Cells were seeded in poly-D-lysine–coated T25 flasks and maintained at 37 °C in a 5% CO_2 incubator, with medium changes every 2–3 days. After approximately 7 days, once confluency was reached, flasks were placed on an orbital shaker (220 rpm, overnight, 37 °C) to remove loosely adherent microglia and oligodendrocyte precursor cells, yielding an enriched astrocyte population.

Single-Nuclei RNA Sequencing (snRNA-seq)

Neocortical tissues were dissected from P7 WT and GS-cKO mice, snap-frozen in liquid nitrogen, and processed for nuclei isolation. snRNA-seq libraries were prepared using the SeekOne® Digital Droplet Single Cell 3' Library Kit (SeekGene, Cat. No. K00202). Reverse transcription, cDNA amplification, adapter ligation (fragmented, end repaired, A-tailed and ligated to sequencing adaptor), PCR amplification, and library purification were performed per manufacturer guidelines. Libraries were sequenced on an Illumina NovaSeq 6000 platform (PE150 mode).

Raw sequencing reads were processed using SeekSoulTools (v1.2.0) with default

parameters. The mouse reference genome GRCm39 (Ensembl v111) was used for alignment. Filtered count matrices were imported into Scanpy (v1.9.8) (Wolf et al., 2018) for downstream analysis. Data were normalized (total count normalization), log1p-transformed, and batch-corrected using Harmony (v0.0.9). Principal component analysis (PCA) reduced dimensionality, and a neighborhood graph was constructed for Leiden clustering (resolution=1.0). Cell types were annotated using canonical marker genes.

Differentially expressed genes (DEGs) in astrocytes (*padj* <0.05, |FoldChange| >1.5) and excitatory neurons (*padj* <0.05, |FoldChange| >1.3) were analyzed using the clusterProfiler package (v4.12.2) (Yu et al., 2012). Gene Ontology (GO) and Kyoto Encyclopedia of Genes and Genomes (KEGG) pathways were enriched with Benjamini-Hochberg-adjusted *p*-values. Top significant terms were visualized as dot plots.

Quantification and statistical analysis

For quantification, position-matched brain sections from control and experimental groups were analyzed, with a minimum of three corresponding sections per group. Fluorescence images were acquired using a Leica TCS SP8 STED microscope equipped with 40× or 60× objectives, using 1–2 μm z-steps and a resolution of 1,024 × 1,024 pixels.

Regions of interest (ROIs) in the somatosensory cortex were defined as 100 μm-wide columns extending from the ventricular surface to the pial surface. Neuronal numbers were quantified by counting marker-positive cells within a 100 μm area in either deep (V–VI) or superficial (II–IV) cortical layers. Cortical neurogenesis in GS-cKO mice was assessed using similarly defined ROIs. Synaptic protein intensity was measured using ImageJ/Fiji, with mean fluorescence intensity calculated following background subtraction. Astrocyte morphology was analyzed with Imaris Bitplane software. Astrocytes with somata positioned within the z-axis range were selected for 3D surface rendering and color-coded based on surface area. Morphological parameters, including filament number and Sholl intersections, were extracted using the Imaris Filament module.

Statistical analyses were performed using GraphPad Prism (version 9.5). Data are presented as mean ± SEM, with replicate numbers (n) indicated in figure legends. Equal variance was assessed using F-tests. Unpaired two-tailed t-tests were used for data with equal variance, while Welch's correction was applied for unequal variance. Multigroup comparisons were analyzed using two-way ANOVA followed by Sidak's or Tukey's post-hoc tests. $P < 0.05$ was considered statistically significant (*$P < 0.05$, **$P < 0.01$, ***$P < 0.001$).

Reference

Hong, W., Gong, P., Pan, X., Ren, Z., Liu, Y., Qi, G., Li, J.L., Sun, W., Ge, W.P., Zhang, C.L., *et al.* (2024). Temporal-spatial Generation of Astrocytes in the Developing Diencephalon. Neurosci Bull 40, 1-16.

Kim, J.Y., Grunke, S.D., Levites, Y., Golde, T.E., and Jankowsky, J.L. (2014). Intracerebroventricular viral injection of the neonatal mouse brain for persistent and widespread neuronal transduction. J Vis Exp, 51863.

Qin, S., Liu, M., Niu, W., and Zhang, C.L. (2011). Dysregulation of Kruppel-like factor 4 during brain development leads to hydrocephalus in mice. Proc Natl Acad Sci U S A 108, 21117-21121.

Wolf, F.A., Angerer, P., and Theis, F.J. (2018). SCANPY: large-scale single-cell gene expression data analysis. Genome Biol 19, 15.

Yu, G., Wang, L.G., Han, Y., and He, Q.Y. (2012). clusterProfiler: an R package for comparing biological themes among gene clusters. OMICS 16, 284-287.

Table 1. Antibodies and Dilution.

Antibodies	Source	Dilution(v/v)
Rabbit anti-Pax6	Oasisbiofarm	1:500
Goat anti-Sox2	Santa Cruz, Chemicon	1:200
Rabbit anti-Tbr2	Abcam	1:300
Mouse anti-Satb2	GeneTex	1:200
Rabbit anti-Brn2	GeneTex	1:200
Rat anti-Ctip2	Abcam	1:500
Rabbit anti-Tbr1	Abcam	1:400
Rabbit anti-SOX9	Sigma-Aldrich	1:500
Mouse anti-ALDH1L1L	Oasisbiofarm	1:500
Rabbit anti-Olig2	Millipore	1:500
Rabbit anti-BLBP	Abcam	1:500
Rabbit anti-S100β	Abcam	1:1000
Mouse anti-GS	Sigma-Aldrich	1:5000
Rat anti-GLAST	Oasisbiofarm	1:300
Chicken anti-GFP	Abcam, AVES	1:1000
Chicken anti-Nestin	Abcam，Covance	1:1500
Rabbit anti-S6 (for Western Blot)	Cell Signaling Technology	1:2000
Rabbit anti-AKT (for Western Blot)	Cell Signaling Technology	1:2000
Rabbit anti-4EBP1 (for Western Blot)	Cell Signaling Technology	1:1000
Rabbit anti-phosphor-S6(for Western Blot)	Cell Signaling Technology	1:1000
Rabbit anti-phosphor-AKT(for Western Blot)	Cell Signaling Technology	1:1000
Mouse anti-β-actin (for Western Blot)	Santa Cruz	1:8000
Alexa Fluor®488 AffiniPure Donkey Anti-IgG (Rabbit, Mouse, Chicken, Rat, Goat)	The Jackson Laboratory	1:500
Alexa Fluor®594 AffiniPure Donkey Anti-IgG (Rabbit, Mouse, Chicken, Rat, Goat)	The Jackson Laboratory	1:500
Alexa Fluor®647 AffiniPure Donkey Anti-IgG (Rabbit, Mouse, Chicken, Rat, Goat)	The Jackson Laboratory	1:500
HRP-anti-Rabbit (for Western Blot)	The Jackson Laboratory	1:5000
HRP-anti-Mouse (for Western Blot)	The Jackson Laboratory	1:5000

Table 2. Primers (In situ hybridization, PCR, RT-qPCR)

Name	Sequences
**In situ hybridization**	
iGS-Forward	ACCTCCATCCTGTTGCCA
iGS-Reverse	ACCGCATAGGGGTCACAA
**PCR**	
Cre-Forward	GCATTACCGGTCGATGCAACGAGTG
Cre-Reverse	GAACGCTAGAGCCTGTTTTGCACGTTC
GS-Forward	GGCTTTAAACCCCTTGAAAGC
GS-Reverse	GATGCCTTTGTTCAAGTGGG
**RT-qPCR**	
qβ–actin-Forward	GGTCATCACTATTGGCAACG
qβ-actin-Reverse	ACGGATGTCAACGTCACACT
qGrm5--Forward	GATCTGTGTGCAGTGAACCGTG
qGrm5-Reverse	CACGCCTTGCAGGTGTACTCAT
qSlc1a4-Forward	GAGGGAGAAGACCTCATCCGAT
qSlc1a5Reverse	GTCACCAGCATGACGATGTCCT
qRac1-Forward	GGACACCATTGAGAAGCTGAAGG
qRac1-Reverse	GTCTTGAGTCCTCGCTGTGTGA
qLrrk2-Forward	GTCAGATGCGCTGGCAAAGCT
qLrrk2-Reverse	AACTCAGTCGGCACAGCTTTCC
qKcnc3-Forward	GAAGAGGTGATTGAAACCAACAGG
qKcnc3-Reverse	TGGGCTCTTGTCTTCTGGAGAC
qAtp6v1g2-Forward	GGAACAGGAGTTTCAGAGCAAGC
qAtp6v1g2-Reverse	TTCCTCTGCTGGGAACTCTGCA
qPrr5-Forward	GGTCCTTCTTCACTGAGTACCTG
qPrr5-Reverse	GAAATCCCAGGTCTCCGCCAAT
qAtp6v1c1-Forward	GGTTGGCTTGTCGGATGAACTG
qAtp6v1c1-Reverse	TGTCCTCCAGCACATCAGCCAT
qAtp6v1c2-Forward	TGGCTCAAGGTGAACTTCAGCG
qAtp6v1c2-Reverse	TGTAGGAGCACAGCCTGGAAGT
qEif4b-Forward	GGACAGGAAGTGAGTCATCGCA
qEif4b-Reverse	GGCTTAGAGGTTGGAGAGTGAC
qHepacam-Forward	GTCTACCTGCTTCTCATCCAGC
qHepacam-Reverse	TTGTCGCTGCTGGTGCTACTGT

Supplemental Fig.S1 Spatiotemporal regulation of glutamine synthetase controls cortical development

(A) Representative images of the cortex stained for GS (red) at P14 and P28. (Scale bars: 200 μm).

(B) Representative images of GS (red) co-stained with S100β, Olig2, IBA1, or NeuN (green) in the cortex at P28. (Scale bars: 20 μm).

(C) Quantification of the proportion of double-labeled cells among total GS+ cells (n = 3 brains).

(D) Quantification of GS activity in the cerebral cortex across developmental stages, including E14, E16, E18, and postnatal days P1, P3, P7, P14, P28, and P60 (n ≥ 3 brains).

(E) Quantification of GS activity in the cerebral cortex of WT and GS-cKO mice at P1, P7, and P14 (n ⩾ 3 brains).

(F) Western blot analysis of GS expression in the forebrain of WT and GS-cKO mice at P7.

(G) Immunofluorescence staining of GS (red) in WT and GS-cKO mice at P28. (Scale bars: 1 mm).

(H) Representative whole-mount images of P14 WT and GS-cKO mouse brains. Scale bars: 5 mm.

(I) Quantification of the projected cortical area in P14 WT and GS-cKO mice (n = 4 mice).

(J) Nissl staining of coronal sections from P14 brains. Red dashed outlines indicate regions enlarged on the right. Scale bars: 1 mm (left); 20 μm (right).

(K) Quantification of cortical thickness in P14 WT and GS-cKO mice (n ≥ 5 mice). Statistical significance was determined using an unpaired two-tailed Student's *t*-test. **P* < 0.05, ***P < 0.001; ns, not significant.Nuclei were counterstained with Hoechst 33342 (Ho; blue).

Supplemental Fig. S2 Deletion of GS does not affect neurogenesis

(A) Representative images of E14.5 mouse cortices stained for EdU (red, labeled 2 h) in WT and GS-cKO mice. (Scale bars: 50 μm).

(B) Quantification of EdU^+ cells per 100-μm ventricular surface width (n = 3 mice).

(C) Representative images of E14.5 mouse cortices stained for Pax6 (white) in WT and GS-cKO mice. (Scale bars: 50 μm).

(D) Quantification of $Pax6^+$ cells per 100-μm ventricular surface width (n = 3 mice).

(E) Representative images of E14.5 mouse cortices stained for Tbr2 (green) in WT and GS-cKO mice. (Scale bars: 50 μm).

(F) Quantification of $Tbr2^+$ cells per 100-μm ventricular surface width (n = 3 mice).

(G) Representative images of P14 cortices stained for Tbr1 (red) and Ctip2 (green) in WT and GS-cKO mice. (Scale bars: 200 μm).

(H) Representative images of P14 cortices stained for Brn2 (red) and Satb2 (green) in WT and GS-cKO mice. (Scale bars: 200 μm).

(I) Quantification of $Tbr1^+$, $Ctip2^+$, $Brn2^+$, and $Satb2^+$ neurons per 100-μm cortical column in WT and GS-cKO mice (n = 3 mice).

Statistical significance was determined using an unpaired two-tailed Student's t-test; ns,not significant.
Nuclei were counterstained with Hoechst 33342 (Ho; blue).

Supplemental Fig.S3 GS-cKO mouse cortex exhibits pronounced astrocyte activation at P28.
(A) Schematic diagram and experimental timeline for selective astrocyte labeling in the postnatal cortex.
(B) Representative images of GFAP immunofluorescence in the cortex of P28 WT and GS-cKO mice.
(C) Quantification of GFAP intensity indices in P28 WT and GS-cKO mice ($n \geq 4$ mice).
Statistical analysis was performed using an unpaired two-tailed Student's t-test. ***$P < 0.001$. Nuclei were counterstained with Hoechst 33342 (Ho; blue).

Supplemental Fig. S4 L-glutamine supplementation alleviates astrocytic maturation defects and synaptic abnormalities in GS-cKO mice
(A) Representative S100β immunofluorescence images showing astrocytic morphology in P28 WT, GS-cKO, and GS-cKO + L-glutamine mice. Arrows denote branched astrocytes; asterisks indicate less branched astrocytes. Scale bars: 25 μm.
(B,C) Quantitative morphological analysis of astrocytes based on total process length (B) and Sholl analysis (C) ($n \geq 20$ astrocytes per genotype from 3 mice).
(D) Golgi-Cox–stained coronal sections of the cortex from P28 WT and GS-cKO mice. Red boxes indicate regions shown at higher magnification on the right. Scale bars: 200 μm.
(E) Representative immunofluorescent images of excitatory synapses (VGLUT1) in layer II/III of the cortex from P28 WT, GS-cKO, and GS-cKO + L-glutamine mice. Scale bars: 50 μm.
(F) Quantification of VGLUT1 intensity indices across groups ($n \geq 3$ mice).
(G) Representative immunofluorescent images of inhibitory synapses (VGAT) from P28 WT, GS-cKO, and GS-cKO + L-glutamine mice. Scale bars: 50 μm.
(H) Quantification of VGAT intensity indices ($n = 3$ mice).
Statistical analyses were performed using one-way ANOVA with Tukey's post hoc test (F, H), an unpaired two-tailed Mann–Whitney test (B), or two-way ANOVA with Sidak's post hoc test (C). *$P < 0.05$, **$P < 0.01$, ***$P < 0.001$, ****$P < 0.0001$; ns, not significant. Nuclei were counterstained with Hoechst 33342 (Ho; blue).

Supplemental Fig. S5 GS-cKO mice exhibit motor function and social interaction deficits
(A) Representative movement traces of WT and GS-cKO mice in the open-field arena.
(B–D) Quantification of total activity time (B), number of center entries (C), and time spent in the center (D) during the open-field test (WT: $n = 12$ mice; GS-cKO: $n = 18$ mice).

(E) Time spent in self-grooming over 15 minutes (WT: *n* = 10 mice; GS-cKO: *n* = 10 mice).
(F) Quantification of total duration of direct social interaction measured by nose-to-body sniffing (WT: *n* = 6 mice; GS-cKO: *n* = 8 mice).
(G–H) Latency to fall (G) and corresponding speed (H) during the rotarod test (WT: *n* = 10 mice; GS-cKO: *n* = 9 mice).
Statistical significance was determined using an unpaired two-tailed Student's *t*-test (B–E) or an unpaired two-tailed Mann-Whitney test (F,G,H). $^{*}P < 0.05$, $^{**}P < 0.01$, $^{***}P < 0.001$, $^{****}P < 0.0001$; ns, not significant.

Supplemental Fig. S6 GS deficiency alters astrocyte marker expression and reduces morphological complexity
(A) Representative images of TUNEL staining in P14 WT and GS-cKO mouse cortices. (Scale bars: 25 μm).
(B) Quantification of the proportion of $TUNEL^{+}$ apoptotic cells in the cortex at P14 (n = 3 mice).
(C) Schematic illustrating the cortical region where GS is highly expressed.
(D) Representative images of P1 cortical sections stained for Blbp/GLAST (red) and GS (green) in wild-type and GS-cKO mice. White rectangles indicate regions shown at higher magnification in D1-D8. (Scale bars: 200 μm; 20 μm for D1-D8).
(E) Quantification of the $GLAST^{+}$ area in the cortex at P1 (n = 4 mice).
(F) Quantification of $Blbp^{+}$ cell numbers in the cortex at P1, P3, and P7 (n ≥ 4 mice).
(G) Dot plot showing the expression patterns of selected astrocyte cluster–specific marker genes. Dot size represents the proportion of nuclei expressing each gene; color intensity indicates average expression level.
(H) Comparison of *Blbp* gene expression in astrocytes between WT and GS-cKO mice (WT, n = 546 cells; GS-cKO, n = 692 cells).
(I) Immunofluorescence and in situ hybridization of *Blbp* in P7 WT and GS-cKO cortices. Arrowheads indicate complex astrocyte branching in WT, while asterisks mark sparse branches in GS-cKO (Scale bars: 50 μm).
(J) Comparison of *ApoE* gene expression in astrocytes between WT and GS-cKO mice (WT, n = 546 cells; GS-cKO, n = 692 cells).
(K) Representative in situ hybridization images of *ApoE* mRNA in P7 WT and GS-cKO cortices. Red rectangles indicate regions shown at higher magnification in F1-F4 (Scale bars: 50 μm).
(L) Quantification of *ApoE* intensity in the cortex at P7 (n ≥ 3 mice).
(M) Comparison of *GLAST* gene expression in astrocytes between WT and GS-cKO mice (WT, n = 546 cells; GS-cKO, n = 692 cells).
(N) Representative in situ hybridization images of *GLAST* mRNA in P7 WT and GS-cKO cortices. Red boxes denote regions magnified in N1–N4. (Scale bars: 50 μm).
(O) Quantification of *G*LAST intensity in the cortex at P7 (n ≥ 3 mice).
Statistical significance was determined using an unpaired two-tailed Student's t-test. $^{*}P < 0.05$, $^{**}P < 0.01$, $^{***}P < 0.001$, $^{****}P < 0.0001$; ns, not significant. Nuclei were counterstained with Hoechst 33342 (Ho; blue).

Figure S1

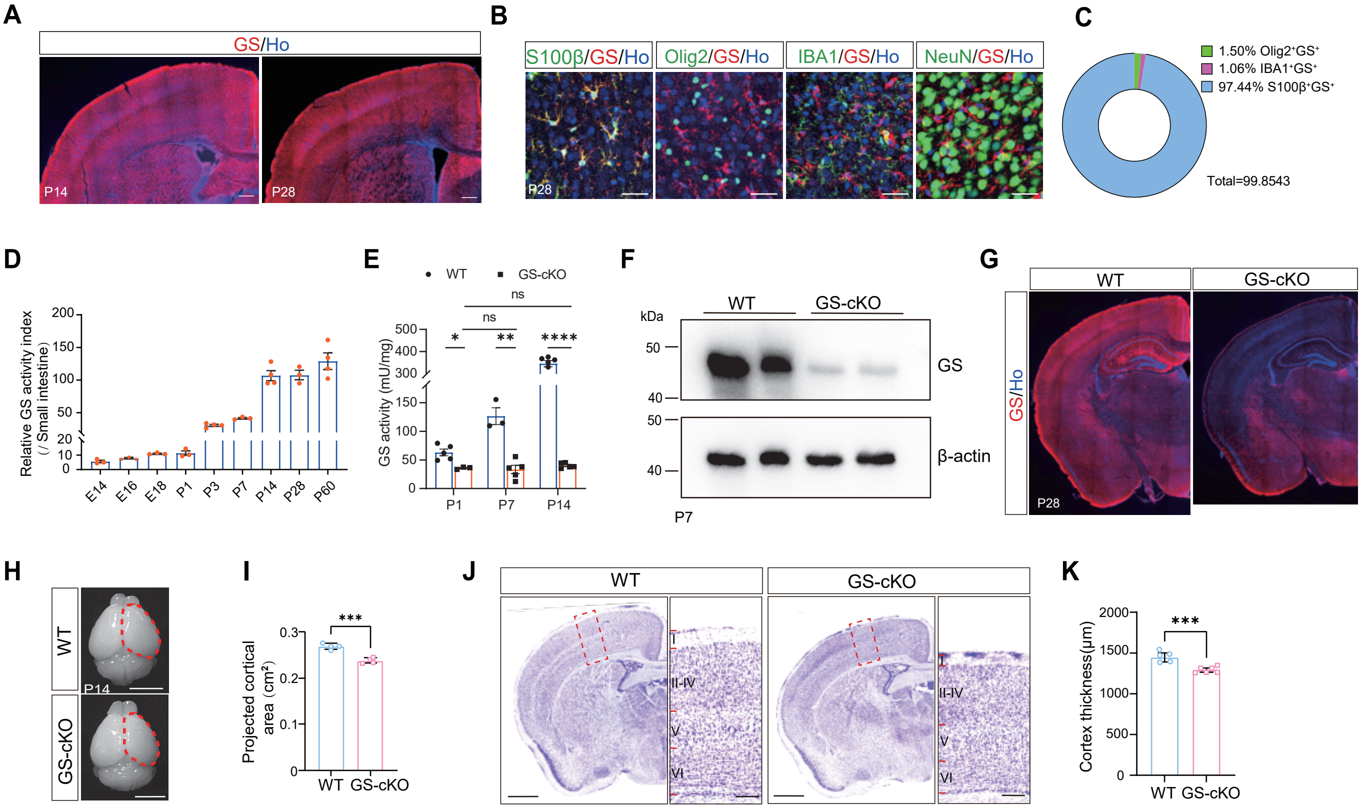

A
GS/Ho
P14
P28
B
S100β/GS/Ho
Olig2/GS/Ho
IBA1/GS/Ho
NeuN/GS/Ho
P28
C
1.50% Olig2+GS+
1.06% IBA1+GS+
97.44% S100β+GS+
Total=99.8543
D
Relative GS activity index (/ Small intestine)
E14 E16 E18 P1 P3 P7 P14 P28 P60
E
WT GS-cKO
GS activity (mU/mg)
ns
ns
*
**
****
P1 P7 P14
F
kDa
WT GS-cKO
GS
β-actin
P7
G
WT GS-cKO
GS/Ho
P28
H
WT
GS-cKO
P14
I
Projected cortical area (cm²)
***
WT GS-cKO
J
WT GS-cKO
I
II-IV
V
VI
K
Cortex thickness(μm)
***
WT GS-cKO

Figure S2

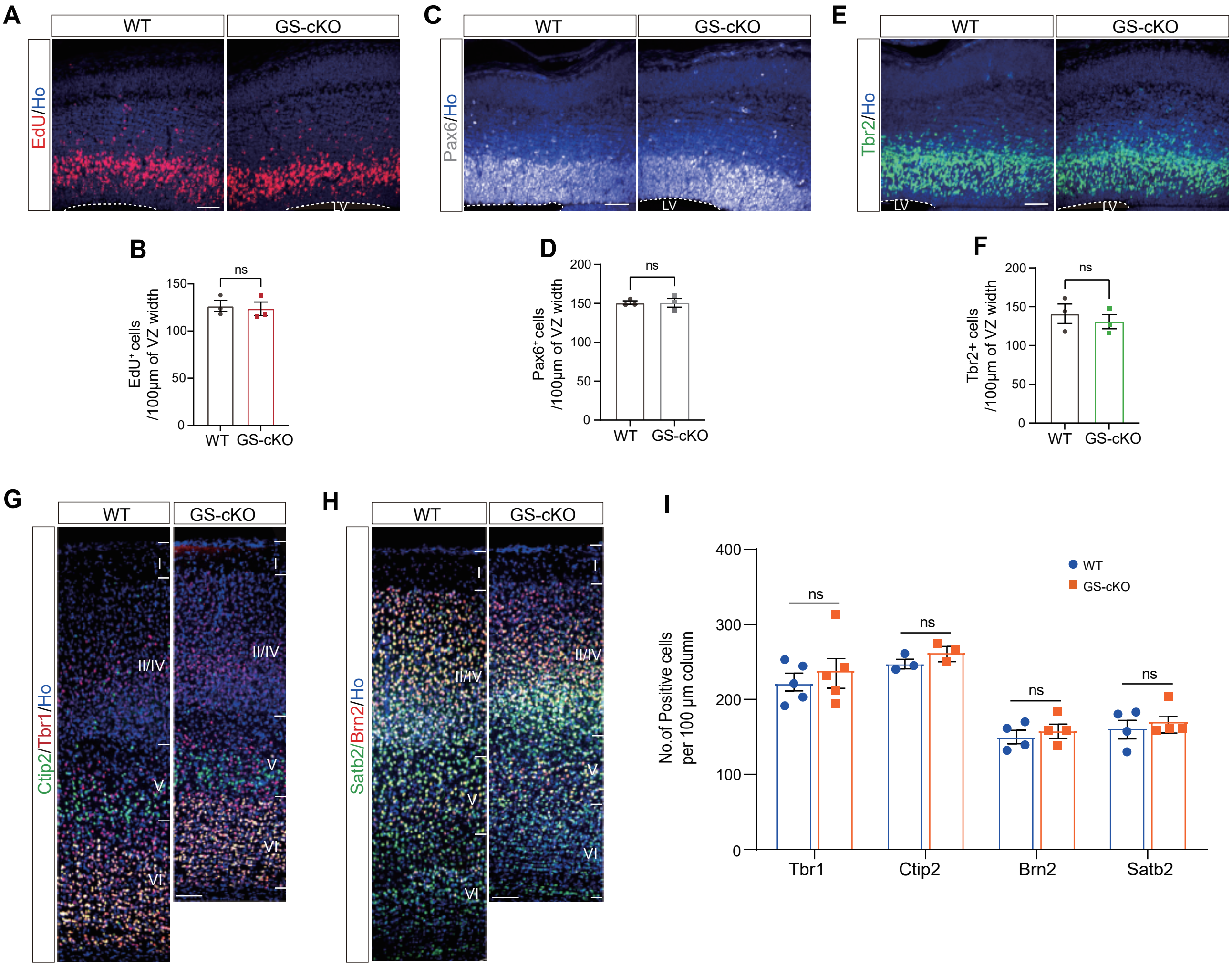

A
WT
GS-cKO
EdU/Ho
LV
C
Pax6/Ho
E
Tbr2/Ho
B
EdU+ cells /100µm of VZ width
ns
D
Pax6+ cells /100µm of VZ width
F
Tbr2+ cells /100µm of VZ width
G
Ctip2/Tbr1/Ho
I
II/IV
V
VI
H
Satb2/Brn2/Ho
No.of Positive cells per 100 µm column
Tbr1
Ctip2
Brn2
Satb2

Figure S3

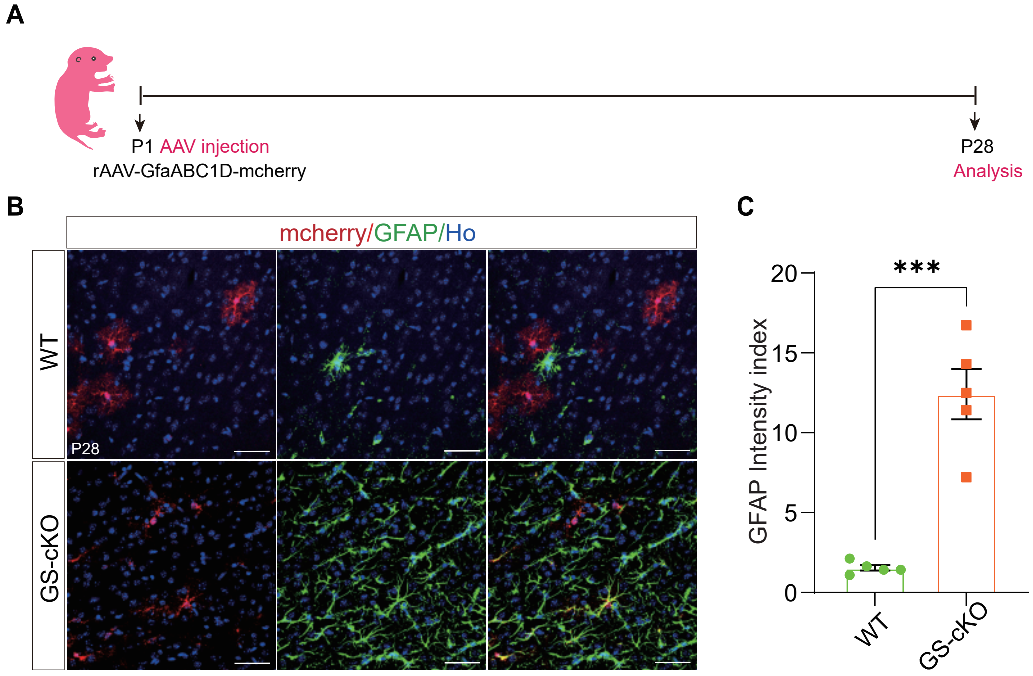
A
P1 AAV injection
rAAV-GfaABC1D-mcherry
P28
Analysis
B
mcherry/GFAP/Ho
WT
GS-cKO
P28
C
***
GFAP Intensity index
20
15
10
5
0
WT
GS-cKO

Figure S4

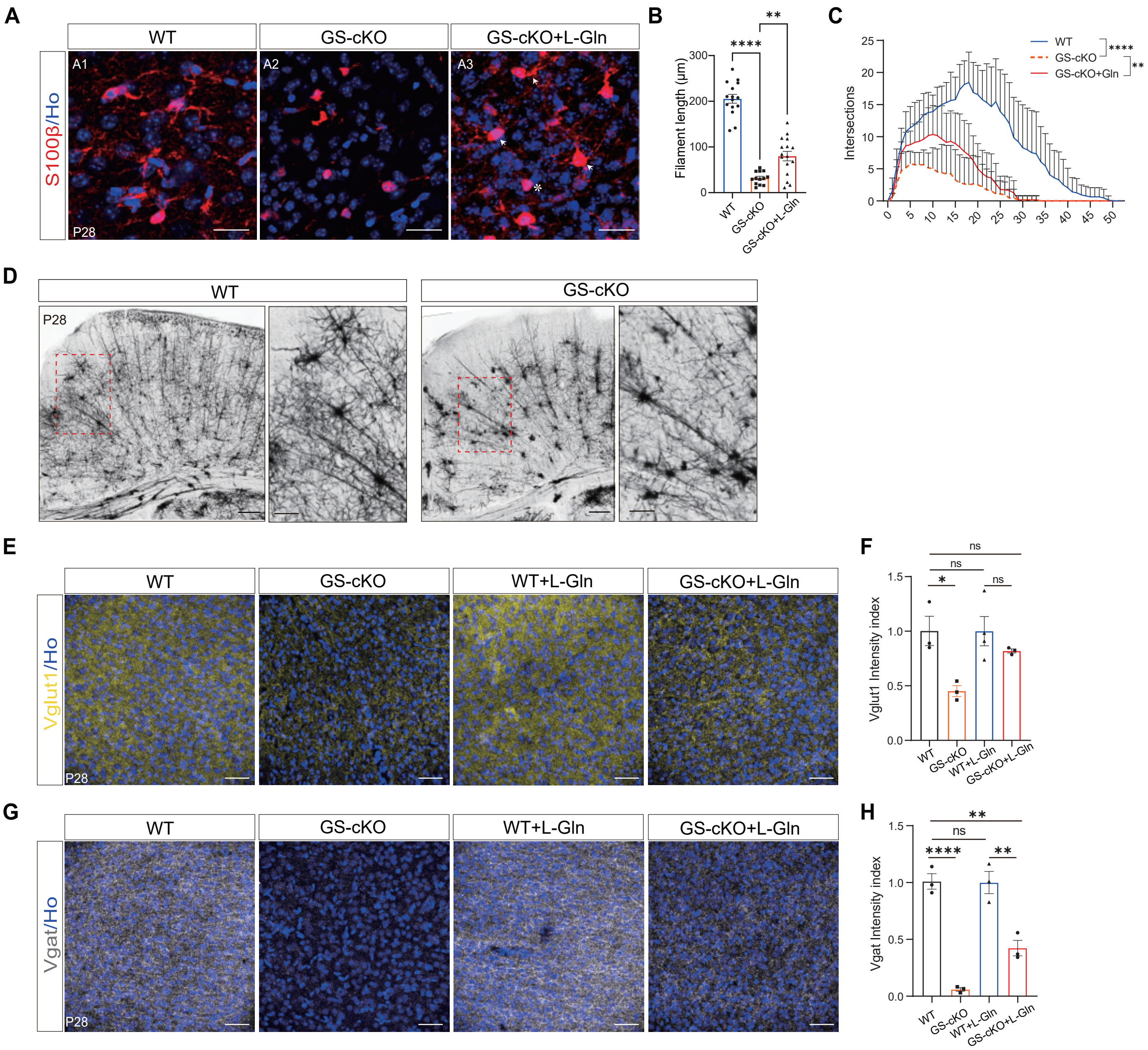
A
WT
GS-cKO
GS-cKO+L-Gln
S100β/Ho
A1
A2
A3
P28
B
Filament length (μm)
300
200
100
0
****
**
WT
GS-cKO
GS-cKO+L-Gln
C
Intersections
WT
GS-cKO
GS-cKO+Gln
****
**
D
WT
GS-cKO
P28
E
WT
GS-cKO
WT+L-Gln
GS-cKO+L-Gln
Vglut1/Ho
P28
F
Vglut1 Intensity index
ns
ns
*
ns
G
WT
GS-cKO
WT+L-Gln
GS-cKO+L-Gln
Vgat/Ho
P28
H
Vgat Intensity index
**
ns
****
**

Figure S5

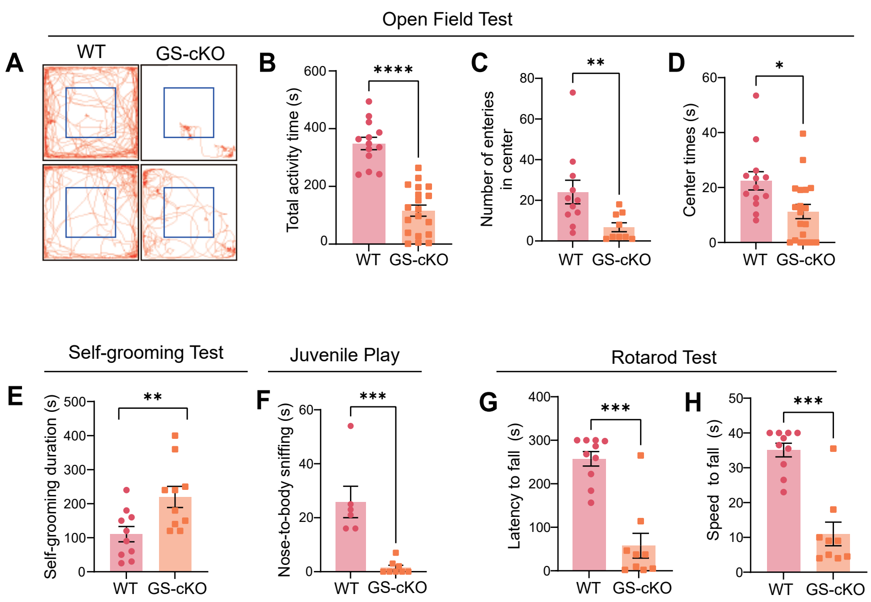
Open Field Test
A
WT
GS-cKO
B
Total activity time (s)
****
C
Number of enteries in center
**
D
Center times (s)
*
Self-grooming Test
E
Self-grooming duration (s)
**
Juvenile Play
F
Nose-to-body sniffing (s)
***
Rotarod Test
G
Latency to fall (s)
***
H
Speed to fall (s)
***
WT
GS-cKO

Figure S6

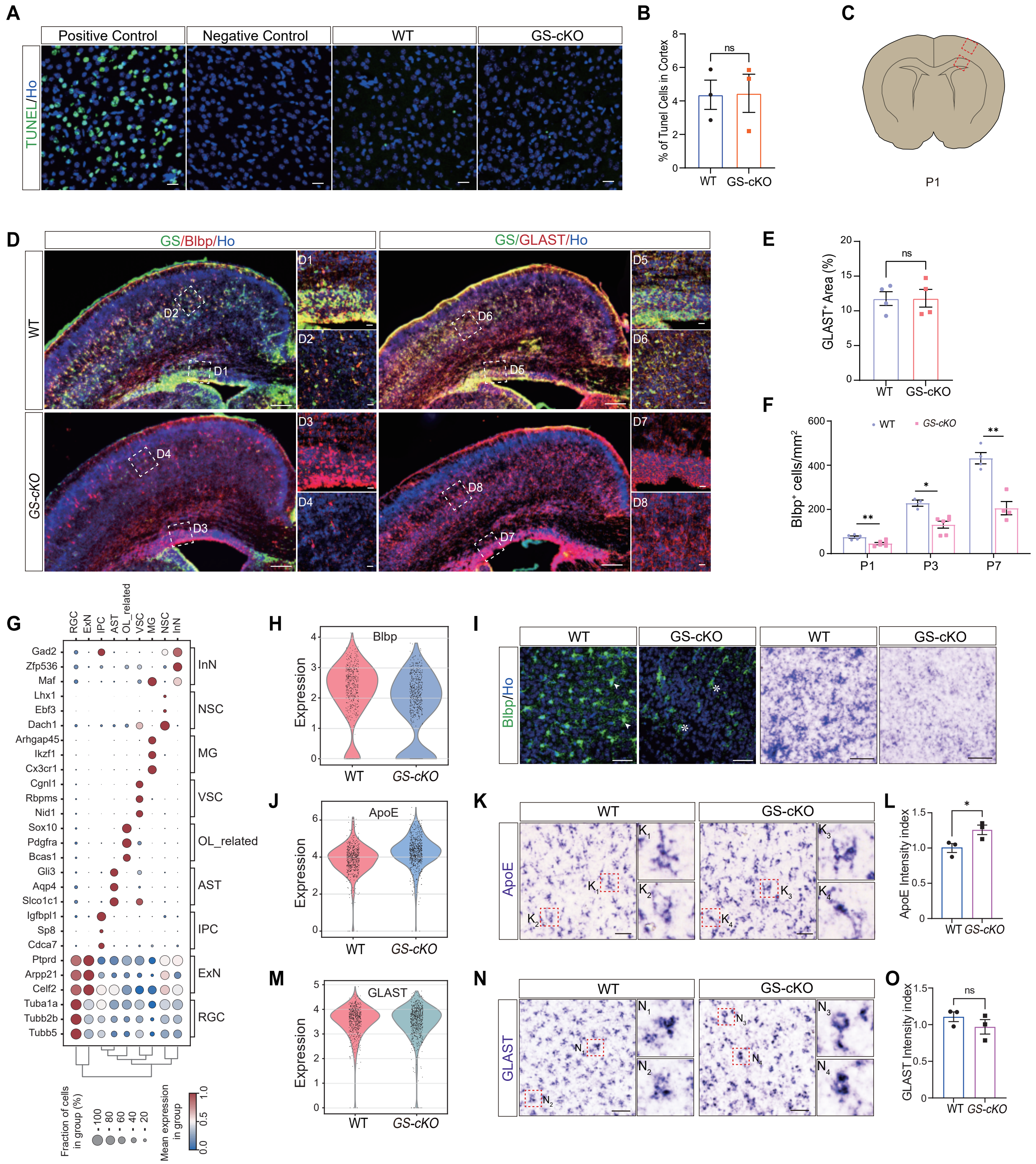

A
Positive Control
Negative Control
WT
GS-cKO
TUNEL/Ho
B
% of Tunel Cells in Cortex
ns
WT
GS-cKO
C
P1
D
GS/Blbp/Ho
GS/GLAST/Ho
WT
GS-cKO
E
GLAST+ Area (%)
ns
F
Blbp+ cells/mm2
P1
P3
P7
G
InN
NSC
MG
VSC
OL_related
AST
IPC
ExN
RGC
Fraction of cells in group (%)
Mean expression in group
H
Blbp
Expression
I
Blbp/Ho
J
ApoE
K
L
ApoE Intensity index
M
GLAST
N
O
GLAST Intensity index
